# Benzbromarone as adjuvant therapy for cystic fibrosis lung disease: a pilot clinical trial

**DOI:** 10.36416/1806-3756/e20230292

**Published:** 2024-05-29

**Authors:** Frederico Friedrich, Lucas Montiel Petry, Laura de Castro e Garcia, Marina Puerari Pieta, Amanda da Silva Meneses, Luana Braga Bittencourt, Luiza Fernandes Xavier, Marcos Otávio Brum Antunes, Lucas Kich Grun, Magali Lumertz, Karl Kunzelmann, Leonardo Araujo Pinto

**Affiliations:** 1. Escola de Medicina, Pontifícia Universidade Católica do Rio Grande do Sul - PUCRS - Porto Alegre (RS) Brasil.; 2. Laboratório de Imunobiologia, Pontifícia Universidade Católica do Rio Grande do Sul - PUCRS - Porto Alegre (RS) Brasil.; 3. Physiological Institute, University of Regensburg, Regensburg, Germany.

**Keywords:** Cystic fibrosis/therapy, Mucociliary clearance, respiratory tract diseases

## Abstract

**Objective:**

Cystic fibrosis (CF) affects multiple organs, the most severe consequences being observed in the lungs. Despite significant progress in developing CF transmembrane conductance regulator-specific treatments for CF lung disease, exploring alternative CF-targeted medications seems reasonable. We sought to evaluate the potential beneficial effects of oral benzbromarone as an adjuvant therapy in CF patients with reduced lung function.

**Methods:**

This was a prospective open-label pilot study of oral benzbromarone (100 mg/day) administered once daily for 90 days. Patients were followed at a tertiary referral center in southern Brazil. Safety was assessed by the number of reported adverse events. Secondary objectives included percent predicted FEV_1_ (FEV_1_%) and pulmonary exacerbations.

**Results:**

Ten patients were enrolled. Benzbromarone was found to be safe, with no serious drug-related adverse events. Eight patients completed the study; the median relative change in FEV_1_% tended to increase during the treatment, showing an 8% increase from baseline at the final visit. However, a nonparametric test showed that the change was not significant (p = 0.06). Of a total of ten patients, only one experienced at least one pulmonary exacerbation during the study.

**Conclusions:**

Oral benzbromarone appears to be safe, and improved FEV_1_% has been observed in patients with CF. Further assessment in larger trials is warranted to elucidate whether oral benzbromarone can be a potential adjuvant therapy for CF.

## INTRODUCTION

Cystic fibrosis (CF) is a severe autosomal recessive genetic disease that is most commonly observed in White populations.^1^ CF affects multiple systems, impacting the function of nearly all body organs by altering the activity of exocrine glands, resulting in reduced function of the CF transmembrane conductance regulator (CFTR) protein.^2^ In some countries, the estimated incidence of CF ranges from approximately 1 per 3,000 live births to 1 per 6,000 live births.^3^ In Brazil, the incidence of CF is approximately 1 per 7,576 live births.^4^

Airway inflammation caused by mucus hypersecretion is the primary contributor to CF morbidity and mortality, with approximately 90% of patients succumbing to the progression of lung disease.^1^ Despite significant progress in developing CFTR-specific treatments for CF lung disease, exploring alternative drug targets in CF appears justified. Several experimental studies have emphasized the role of TMEM16A in mucus production and secretion.^5^ ^-^ ^8^ TMEM16A is an alternative calcium-activated chloride channel contributing to mucus hypersecretion and bronchoconstriction.^9^ Benzbromarone is a drug that has been shown to inhibit TMEM16A activity in animal models.^8^ ^,^ ^10^ ^,^ ^11^ Recent evidence suggests that inhibiting TMEM16A function may offer therapeutic benefits, potentially reducing mucus production and impacting the severity of CF lung disease.^12^

Oral benzbromarone is a potent uricosuric agent indicated for the treatment of chronic gout.^13^ It has been approved for use in Brazil by the Brazilian Health Regulatory Agency and has been commercially available since the 1970s.^14^ Given its low cost, easy accessibility, and ability to control the activity of CF-related ion channels, benzbromarone is an intriguing alternative for the treatment of CF irrespective of the presence of disease-causing mutations. 

On the basis of experimental models,^5^ ^,^ ^6^ ^,^ ^9^ ^-^ ^11^ we conducted a pilot clinical trial to evaluate the potential beneficial effects of oral benzbromarone (100 mg/day) as an adjuvant therapy in CF patients with reduced lung function. 

## METHODS

### Study design, participants, and setting

We conducted an open prospective pilot trial involving ten CF patients at a tertiary referral center in southern Brazil. The study was conducted in accordance with the amended Declaration of Helsinki, with approval from local institutional review boards (Protocol no. 3.927.092; CAEE no. 29576220.0.0000.5336). Written informed consent was obtained from all patients and their parents. The trial is registered in Brazil under identifier number RBR-5wvg53s (available at https://ensaiosclinicos.gov.br/rg/RBR-5wvg53s). 

Children > 6 years of age were included if they had a confirmed diagnosis of CF based on the following criteria: a positive sweat chloride test (> 60 mEq/L) and identification of two pathogenic variants in the *CFTR* gene. Additionally, participants had to have a percent predicted FEV_1_ (FEV_1_%) of < 90% in order to be classified as having reduced lung function. Key exclusion criteria included pregnancy, initiation of new therapy, use of CFTR modulators, and experiencing a pulmonary exacerbation within four weeks before the study initiation. 

The primary outcome was the safety of oral benzbromarone, as assessed by pulmonary exacerbations, vital signs, physical examination, hematological markers, coagulation markers, and drug-related adverse events.^15^ Liver function was also assessed,^14^ and if any adverse effects were observed, the drug was discontinued until clarification. 

Spirometry tests (FEV_1_%) were performed at screening, at baseline (day 1), before the first day of treatment with oral benzbromarone, and during follow-up (at 30, 60, and 90 days). Changes in FEV_1_% were assessed by comparing FEV_1_% values obtained on day 1 with those obtained at the final study visit. Spirometry was performed with a KoKo spirometer (KoKo PFT, Longmont, CO, USA), in accordance with the guidelines of the American Thoracic Society and European Respiratory Society.^16^ The results were presented as absolute values and percentages of the predicted values.^17^ The number of pulmonary exacerbations was evaluated during follow-up (at 30, 60, and 90 days). 

### Intervention

All patients received standard CF treatment, including chest physiotherapy, dietary supplementation, fat-soluble vitamins, pancreatic enzyme replacement therapy for pancreatic insufficiency, and antibiotics for CF respiratory exacerbations. Furthermore, patients were instructed to take 100 mg of oral benzbromarone once daily for 90 days. The dose was determined on the basis of the established use of benzbromarone in the treatment of chronic gout. This dose has been shown to be safe and effective in adults with gout, making it a reasonable starting point for exploration in the CF population. 

### Statistical analysis

Given that previous studies have identified a 10% increase in FEV_1_ with the use of ivacaftor, we opted for a more conservative increase. We adopted a significance level of 0.05, a power of 80%, and a standard deviation of 10.0 with the objective of detecting a minimum expected difference of 5% in the values of the variable of interest (FEV_1_). The calculated sample size was 32 individuals. The inclusion of lung function measurements allowed a more comprehensive assessment, aligned with clinical relevance, although safety remained the primary focus. Sample size parameters were chosen to detect a 5% difference, balancing potential clinical impact with safety evaluation. Results were expressed by descriptive statistics (median and interquartile range). Comparisons were made with the paired Wilcoxon test. Statistical analysis was performed with the R computing environment (version 4.2.1, R Foundation for Statistical Computing, Vienna, Austria). 

## RESULTS

**Figure 1:**
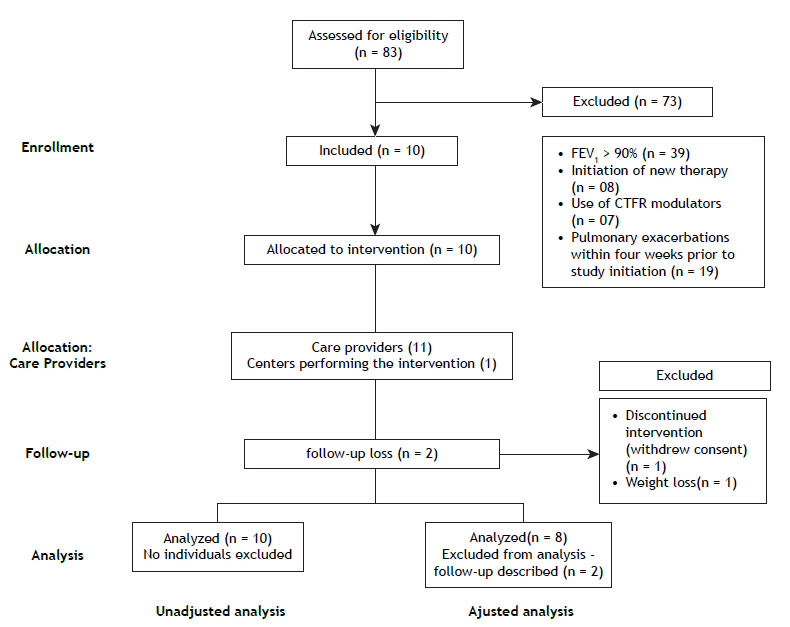
Flow chart of screening and follow-up.

Eighty-three patients with a confirmed diagnosis of CF were evaluated at our center. Of those, ten were eligible for inclusion in the study (Figure 1). Baseline demographics are summarized in Table 1 (unadjusted analysis). Eight patients completed the treatment with oral benzbromarone (adjusted analysis). 

**Table 1:** Demographic characteristics of the participants at baseline.^a^

Characteristic	Result
N (female:male)	10 (4:6)
Age, years	10 (9-16)
Weight, kg	40 (33-47)
Height, cm	141 (139-151)
Meconium ileus at diagnosis/birth	3 (30%)
*CFTR* genotype	
Heterozygous for F508del	7 (70%)
Homozygous for F508del	3 (30%)
Chronic colonization of the airways	
*Staphylococcus aureus*	8 (80%)
*Pseudomonas aeruginosa*	2 (20%)
Pulmonary function	
FEV_1_ (% predicted)	84 (53-88)

a Data presented as n (%) or median (IQR), except where otherwise indicated.

The primary endpoint of this pilot study was the safety of oral benzbromarone in CF patients. No serious drug-related adverse events were reported. Two participants were excluded from the final analysis. The only adverse event identified was weight loss (a loss of 3 kg in one patient), which was not considered severe, because the patient did not use the medication as recommended. Oral benzbromarone was discontinued, and the patient was started on standard treatment, after which the weight loss resolved. The patient continued to be followed at our center. Additionally, one patient chose to withdraw consent and discontinue the treatment with oral benzbromarone. Throughout the study period, no changes were found in patient vital signs or laboratory test results (data not shown). 

**Figure 2:**
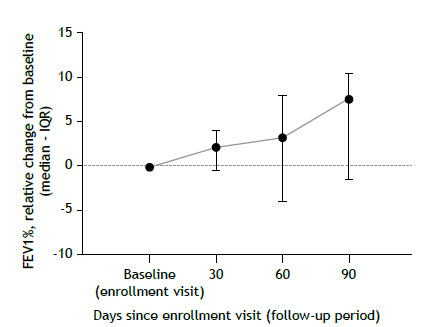
The effect of oral benzbromarone on lung function was assessed by measuring median changes in FEV_1_% from baseline (enrollment visit) on spirometry in eight patients at 30, 60, and 90 days. The paired Wilcoxon test showed no significant change in FEV_1_% (p = 0.06).

The median relative change in FEV_1_% tended to increase during benzbromarone treatment, showing an 8% increase from baseline at the final visit (at 90 days; Figure 2). However, we observed no significant difference in the increase in FEV_1_% (p = 0.06). Furthermore, only one patient experienced one episode of pulmonary exacerbation during the study period. The patient was treated and did not require hospital admission. 

## DISCUSSION

This prospective open-label pilot study was the first clinical trial to assess the use of oral benzbromarone as an adjuvant for treating CF. Oral benzbromarone was found to be safe, with no reports of serious adverse events. Although the study was not powered to achieve statistical significance in secondary outcomes, increases in the overall mean values of FEV_1_% were observed during the 90 days of treatment. 

Our findings are consistent with those of a recent preclinical animal study, suggesting that inhibiting an alternative chloride channel may be an attractive therapeutic strategy in CF.^5^ ^,^ ^8^ ^,^ ^11^ Importantly, because TMEM16A expression and function are independent of disease-causing mutations in the *CFTR* gene, this therapeutic approach is predicted to be suitable for all patients with CF irrespective of their genotype.^18^ The counterintuitive idea of using inhibitors of TMEM16A channels is based on their role in mucus production and secretion, as recently evidenced.^11^ This proposal is grounded in reports that nonselective regulators of TMEM16A function may affect airway smooth muscle and mucin release from goblet cells.^10^ ^,^ ^19^ ^,^ ^20^ Therefore, potent and safe TMEM16A inhibitors should be further examined in preclinical and clinical studies for use in CF lung disease and other inflammatory airway diseases. 

Studies have indicated that airway calcium-activated chloride channels, such as TMEM16A and ETX001, hold potential therapeutic benefits for CF treatment.^19^ However, the numerous physiological attributes associated with the former, such as improved lung function through mucociliary clearance, especially in the main airways, may enhance therapeutic action by increasing the channel activity.^11^

This regulation encompasses airway smooth muscle contraction and airway hyperresponsiveness, along with goblet cell formation and exocytosis.^10^ In fact, TMEM16A expression has been identified in airway smooth muscle, and TMEM16A blockers, such as benzbromarone and niclosamide, have been shown to attenuate smooth muscle contraction in human and murine airways. Additionally, benzbromarone has been shown to impair mucin release from epithelial cells of human airways, suggesting a role in the mechanism of exocytosis.^20^ ^-^ ^22^

Several limitations of our clinical trial should be acknowledged. The primary limitation is the small sample size, which constrained the statistical power to detect differences, with 80% of the sample consisting of individuals < 18 years of age. Consequently, the results should be interpreted with caution. A critical limitation of this study is the absence of a control group. Given the small sample size and variability observed in FEV_1_ measurements, the lack of a control group makes it challenging to conclusively attribute lung function improvement to the intervention. A control group would have provided a baseline for comparison and a more robust assessment of the impact of the intervention. Additionally, the fact that this was an open study conducted at a single center introduces the possibility of bias. To mitigate this, the study team reviewed all data twice, and, in case of discrepancies, a third review was considered. Furthermore, all information was recorded on electronic medical records. 

Another critical point is the small number of adverse events observed in our study. This may be attributed to the low number of participants and the short duration of treatment. It is also of note that we collected no information regarding mucus production in the study participants. This information could contribute to a better understanding of the biological response mechanisms of the proposed therapeutic option. 

Oral benzbromarone appears to be safe, and improved FEV_1_% has been observed in patients with CF. Importantly, because TMEM16A expression and function are independent of disease-causing mutations in the *CFTR* gene, this adjuvant therapeutic option is predicted to be suitable for all patients with CF. This study represents the first evaluation of the use of oral benzbromarone in patients with CF. Further assessment in larger trials is warranted to elucidate whether oral benzbromarone can be a potential adjuvant therapy for CF. 
